# Parametric seasonal-trend autoregressive neural network for long-term crop price forecasting

**DOI:** 10.1371/journal.pone.0311199

**Published:** 2024-09-26

**Authors:** Woojin Hong, Seong Cheon Choi, Seungwon Oh

**Affiliations:** 1 Resource Management Department, Jeonnam Agricultural Research & Extention Services, Naju-si, Jeollanam-do, South Korea; 2 Department of Agricultural Outlook, Korea Rural Economic Institute, Naju-si, Jeollanam-do, South Korea; 3 Artificial Intelligence Institute, Korea University, Seongbuk, Seoul, South Korea; National Institute of Technology Rourkela, INDIA

## Abstract

Crop price forecasting is difficult in that supply is not as elastic as demand, therefore, supply and demand should be stabilized through long-term forecasting and pre-response to the price. In this study, we propose a Parametric Seasonal-Trend Autoregressive Neural Network (PaSTANet), which is a hybrid model that includes both a multi-kernel residual convolution neural network model and a Gaussian seasonality-trend model. To compare the performance of the PaSTANet, we used daily data from the Garak market for four crops: onion, radish, Chinese cabbage, and green onion, and performed long-term price forecasts for one year in 2023. The PaSTANet shows good performance on all four crops compared to other conventional statistical and deep learning-based models. In particular, for onion, the (mean absolute error (MAE) for the long-term forecast of 2023 is 107, outperforming the second-best Prophet (152) by 29.6%. Chinese cabbage, radish, and green onion all outperform the existing models with MAE of 2008, 3703, and 557, respectively. Moreover, using the confidence interval, the predicted price was categorized into three intervals: probability, caution, and warning. Comparing the percentage of classified intervals about the true prices in our test set, we found that they accurately detect the large price volatility.

## 1. Introduction

Forecasting crop prices remains a challenging task as it is influenced by a variety of external features, including weather conditions, growing conditions, yields, and fluctuations in demand [1,2]. These uncertainties can also pose economic risks to both producers and consumers [3,4]. Therefore, long-term forecasting of crop prices is required not only to regulate supply and demand through agricultural production planning and sales planning but also to increase the stability of the agricultural economy and improve the efficiency of agricultural markets [5].

The Korea Rural Economic Institute uses the Korean Agriculture Simulation Model (KASMO) to forecast the supply and demand of 45 agricultural products [6]. KASMO is a regression-based model that considers various variables such as production area, production location, and compost use. However, models with multiple variables have limitations because temporal information is not reflected in the model, and thus not applicable to daily prices. Therefore, a time-series-based price prediction model using daily prices is required.

Crop price, which is time-series data, has three characteristics [7]. First, it contains time-lagged information, implying the data are correlated. Therefore, recent prices contribute more to future predictions than previous prices. Second, the time series displays a constant trend and regular and irregular seasonality. Finally, the real-world time-series data are subject to missing and extreme data. Consequently, various approaches utilize models that reflect these characteristics of time-series data to perform forecasting.

The autoregressive integrated moving average (ARIMA) model, which uses previous prices, is a statistical forecasting model commonly used in time-series analysis. It combines three time-series models: the autoregressive (AR) model, which explains the current value of a variable based on its past values; the moving average model, which explains the current value of a variable based on past forecast errors; and the integrated model, which differentiates the time-series data to remove non-stationarity. Most previous research forecasted prices using ARIMA models [8–11].

Seasonal-trend decomposition using locally estimated scatterplot smoothing (LOESS), namely STL, models data as a time function. STL is a robust non-parametric method for decomposing time-series data into trend, seasonal, and residual components. In contrast to traditional decomposition methods that rely on parametric assumptions, STL utilizes the flexible LOESS smoother to locally estimate the trend and seasonal components [12,13].

In the time-series forecasting process, AR models that use previous prices and STL models that use time functions have advantages and disadvantages because they utilize different features of the time-series data (Table 1). AR has the advantage of providing intuitive interpretation as it relates past prices to future prices, incorporates recent information, and can be combined with various data. However, the appropriate sequence length for historical prices is difficult to determine and is sensitive to extreme and missing values. The STL model has the advantage of being able to make long-term forecasts because of its strong functional assumptions, ability to analyze the components of the time series, and robustness to extraneous and missing values. However, long-term forecasting is trend-dependent, does not reflect recent information, and has difficulty considering exogenous variables.

**Table 1:** Advantages and disadvantages of conventional forecasting methods.

Method	Advantages	Disadvantages
AR	1) Intuitive interpretation is possible 2) Reasonable predictions based on recent information 3) Applicable to various data	1) Difficulty in determining the appropriate lag 2) Sensitive to missing values 3) Sensitive to extreme values
STL	1) Enable long-term forecasting 2) Decomposing the characteristics of time series data 3) Robustness to missing values	1) Dependence on trend predictions 2) Limiting the use of exogenous variables 3) Difficulty incorporating recent information

AR = autoregressive, STL = Seasonal and Trend decomposition using Loess.

The recurrent neural network (RNN), temporal convolution network (TCN), and Transformer models are represented AR models that perform well in time forecasting. First, RNN [14,15] processes sequential data using a hidden state to capture information from previous inputs, and TCN [16] uses causal convolutions and dilation for parallel sequence processing. Lastly, the Transformer uses a self-attention module for connecting between sequence positions and achieving good performance [17]. However, these models are AR-based models, which have the limitation that RNNs have difficulty reflecting long sequences [18], and TCNs may not reflect seasonality at different scales because of the need to determine the appropriate kernel size [19,20]. In addition, Transformer requires high computational resources and has the limitations of hyper-parameterization and complexity.

**Fig 1:**
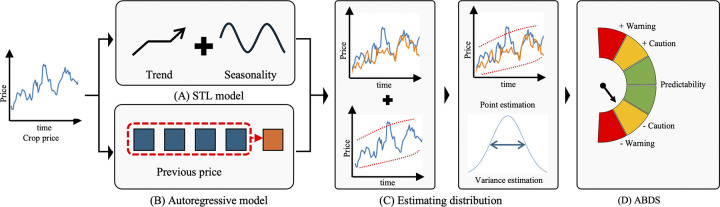
The proposed abnormal price detection system estimates price distribution, considering both time and previous crop prices. (A) Feature extraction from crop price using seasonality-trend by loess (STL). (B) Feature extraction from crop price using autoregressive model. (C) Estimation of *μ* and *σ*^2^ for crop price distribution (*f*(*t*|*μ*, *σ*^2^)). (D) Abnormal price detection system (APDS) using confidence interval.

Therefore, we propose a hybrid approach for forecasting crop prices using both AR and STL modules (Fig 1). The proposed parametric seasonal-trend autoregressive neural network (PaSTANet) model uses the AR method to incorporate the previous price and the time function of the STL method to predict the crop price. Subsequently, the distribution of the predicted price is estimated, and abnormal prices are detected by comparing the estimation with the current price. The contributions of PaSTANet are as follows:

1. Hybrid deep learning models using both STL and AR outperform previous models.
2. The model outputs are the parameters of a Gaussian distribution, allowing point estimation and measurement of uncertainty.
3. Appropriate thresholds are suggested for the crop price, allowing the development of an appropriate response strategy.

## 2. Materials and methods

### 2.1 Data

Daily data on crop prices were obtained from the Garak market of the Seoul Korea Agriculture Fisheries & Food Trade Corporation (aT) (Table 2). In this study, except for radish, data from 2010 to 2022 were used, with the 2023 data as the test dataset. There are two reasons for the difficulty in forecasting crop prices. The first is the requirement for using daily data. With weekly or monthly data, the effect of extreme values is reduced because the period is averaged. However, in the case of daily data, extreme values are inevitable, increasing the sensitivity to human error or specific transactions. Second, missing values do not exist at regular intervals. For example, Saturdays and Sundays occur at regular intervals, so they occur in a five-day cycle. However, excluding the weekends, there are irregular missing values corresponding to holidays with no transactions, and each crop is a seasonal vegetable grown in the open field. To reduce the effects of missing values that cause poor data quality, imputation methods can be used; however, they also have limitations because they are not real data [21,22]. The data can be accessed from the following repository: https://doi.org/10.34740/KAGGLE/DS/5401197.

**Table 2:** Description of crop price (won/kg) in this study.

Crop	Time lag	Sample size	Range	Median	mean±std
Onion	[2010, 2023]	4,280	(300, 2833)	906	957±375
Green onion	[2010, 2023]	4,586	(342, 5825)	1,507	1651±740
Chinese cabbage	[2010, 2023]	3,961	(1536, 30337)	6,846	7462±4098.58
Radish	[2012, 2023]	3,616	(3773, 38448)	11,271	12949±5553

### 2.2 Model

**Fig 2:**
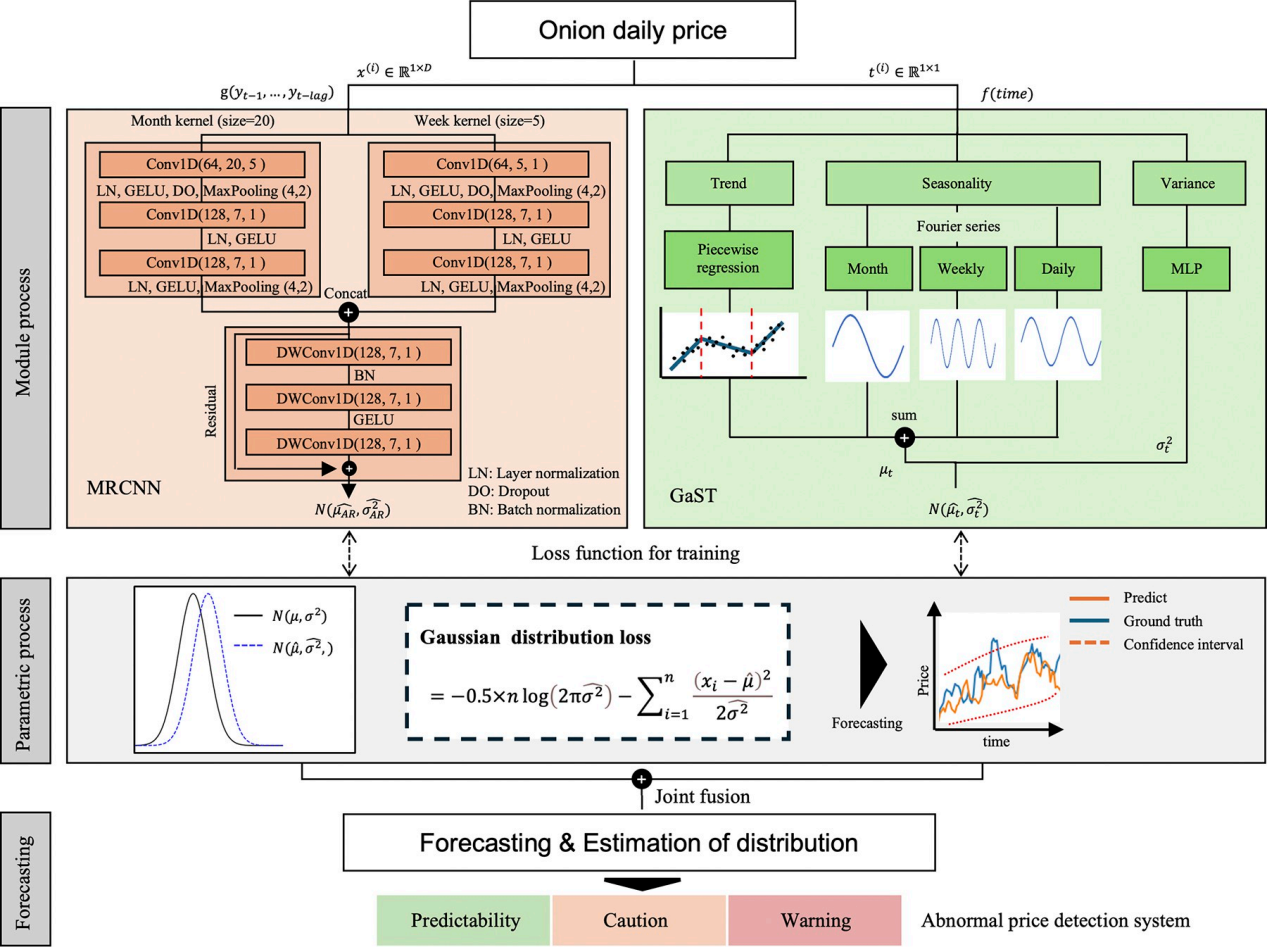
Illustration of the proposed model.

Fig 2 illustrates the PaSTANet method proposed in this study. PaSTANet consists of a multi-kernel residual convolution neural network (MRCNN) module, a Gaussian seasonality-trend (GaST) module, and a parametric process. The MR-CNN module is a CNN-based AR model that uses different kernels to reflect weekly and monthly effects. The GaST module consists of a piecewise-based linear model for trend prediction and Fourier terms to reflect seasonality. Finally, the parametric process estimates the parameters of the Gaussian distribution of the individual models, with joint fusion applied to perform training.

#### 2.2.1 Multi-kernel residual convolution neural network module

The MRCNN module applies the AR method to historical price information. The crop price shows a certain volatility depending on how they are consumed during the growing, harvesting, and storage seasons. Therefore, we considered a multi-kernel CNN to reflect weekly and monthly effects [23,24]. A multi-kernel system consists of a kernel to reflect the weekly effect of five days, excluding weekends, and a kernel to reflect the monthly effect, concatenating the outputs.

The concatenated feature map combines features using three depth-wise CNNs (DWCNNs). Unlike conventional CNNs, the DWCNN is a method of convolving channels by groups and has shown good performance in previous studies [25,26]. The three DWCNNs are stacked using residual connections. As a result, the output of MRCNN is a Gaussian distribution with mean $\hat{\mu_{AR}}$ and standard deviation $\hat{{\sigma_{AR}}^{2}}$.

#### 2.2.2 Gaussian seasonality-trend (GaST) module

The GaST module applies the STL method to learn and combine the trend and seasonality models. It applies piecewise regression and uses the Fourier series of the conventional prophet and neural prophet models [27]. Seasonality considers three types of volatility: monthly, weekly, and daily, further adding a multilayer perceptron (MLP) for $\hat{{{and\;\sigma }_{t}}^{2}}$. As a result, the output of GaST is a Gaussian distribution with mean $\hat{\mu_{t}}$ and standard deviation $\hat{{\sigma_{t}}^{2}}$.

#### 2.2.3 Parametric process

Note that the outputs of MRCNN and GaST are Gaussian distribution parameters. The maximum likelihood estimation (MLE) method was used to learn the respective distribution parameters. MLE is used to determine the parameter that provides the largest likelihood for a sample, which is usually estimated using the log-likelihood. This parametric process is applied to estimate *μ* and *σ*^2^ respectively.

The likelihood function (*L*(*μ*, *σ*^2^)) is used to estimate the parameters of a distribution. A likelihood function measures the likelihood that a set of observed data can occur given a set of parameters. The likelihood function was used to calculate the maximum likelihood estimates of the parameters, which is the set of parameter values that maximize the likelihood of the observed data. Because we considered the exponential family distribution, the log-likelihood was more convenient. Therefore, we set the loss function (*Loss*) to minimize the negative log-likelihood.


$$L(\mu ,\sigma^{2})=\prod_{i=1}^{n}f(t_{i};\mu ,\sigma^{2})$$ (1)

$$\log\left(L(\mu ,\sigma^{2})\right)=\sum_{i=1}^{n}\log\left(f(t_{i};\mu ,\sigma^{2})\right)$$ (2)

$$Loss=\arg\underset{\mu ,\sigma^{2}}{\min}-\log\left(L(\mu ,\sigma^{2})\right)$$ (3)

The log likelihood (log(*L*(*μ*, *σ*^2^))) for a Gaussian distribution is calculated as follows:

$$Loss=\sum_{i=1}^{n}-\log\left(L(\mu ,\sigma^{2})\right)=\sum_{i=1}^{n}\left(-0.5\times \log\left(2\pi \sigma^{2}\right)-\frac{{\left(t_{i}-\mu \right)}^{2}}{2\sigma^{2}}\right)=-0.5\times n\;\log\left(2\pi \sigma^{2}\right)-\sum_{i=1}^{n}\frac{{\left(t_{i}-\mu \right)}^{2}}{2\sigma^{2}}$$ (4)

As the individual models with losses were considered independently, we adopted the joint fusion method of multimodal fusion [28]. The resulting estimated Gaussian distributions were combined to forecast crop price. The main difference between our method and existing methods is the assumption of a Gaussian distribution for the predicted value, which allows deriving a confidence interval (CI). To apply the forecast results, three intervals were distinguished according to the point estimation of the crop price and CI to help control the supply and demand of the current price.

### 2.3 Evaluation metrics

Four metrics were considered in comparing the performances of the models: mean absolute error (MAE), mean absolute percentage error (MAPE), standard deviation error (SDE), and median absolute error (MedAE). Because the root mean squared error, which is commonly used for prediction error, uses the square root of the square of the error, it can be overestimated for large units. Therefore, we adopted MAE, which uses absolute values for the baselines. A brief explanation of the four metrics is provided below.

1. MAE is the average of the absolute values of the errors without changing units.
2. MAPE is the mean of the error divided by the actual value and is expressed as a percentage.
3. SDE is related to the distribution of errors, allowing the stability of the predicted value to be evaluated.
4. MedAE is the median of the errors and is characterized by robustness to outliers.

$$MAE=\frac{1}{n}\sum_{i=1}^{n}\left|y_{i}-{\hat{y}}_{i}\right|$$ (5)

$$MAPE=\frac{1}{n}\sum_{i=1}^{n}\left|\frac{y_{i}-{\hat{y}}_{i}}{y_{i}}\right|$$ (6)

$$SDE=\sqrt{\frac{1}{n}\sum_{i=1}^{n}{\left(|y_{i}-{\hat{y}}_{i}|-\frac{1}{n}\sum_{i=1}^{n}\left|y_{i}-{\hat{y}}_{i}\right|\right)}^{2}}$$ (7)

$$MedAE=Median(|y_{1}-{\hat{y}}_{1}|,|y_{2}-{\hat{y}}_{2}|,\ldots ,|y_{n}-{\hat{y}}_{n}|)$$ (8)

Here, *y*_*i*_ is i^th^ ground truth value; ${\hat{y}}_{i}$ is i^th^ predicted value.

### 2.4 Experimental setting

For the daily crop price from 2010 to 2023, the data from 2023 were used as the test set. The data from 2010 to 2022 (in the case of radish, 2012 to 2022) were randomly split, with 80% as the training set and 20% as the validation set. A batch size and learning rate of 64 and 5e-4 were used, respectively, to determine through experiments the number of change points and Fourier series for the GaST module and the number of stacks of the residual block. The best performance was achieved with five change points, four Fourier series on GaST, and two stacks of residual blocks on MRCNN.

## 3. Results

This section shows the results of forecasting four crop prices. In particular, we show the overall results for onions because it is the main seasoning vegetable consumed in Korea. The results of the remaining crop prices show the prediction results of the model that performed well in forecasting onion prices.

### 3.1 Comparison of model performance

The performances of conventional and deep-learning-based models were compared. For the conventional method, AR-based ARIMA [29] and STL-based Prophet [30] were compared. The statistical models ARIMA and Prophet model need to determine the hyper-parameters including the order of the model. For all crops, ARIMA used the "auto_arima" function of the "pmdarima v2.0.3" package to determine the hyper-parameters, and for the Prophet model, we let the model determine them automatically, using 25 changepoints, linear growth, additive seasonality mode, 10 yearly Fourier orders, and 3 weekly Fourier orders for the initial values.

For all benchmark deep-learning models, we used “NeuralForecast” packages, which provide a diverse collection of neural network forecasting models focusing on performance, usability, and robustness, and the “NerualProphet” package for the NeuralProphet model. The input size was 2×365, and the output size was 365. The maximum number of steps for training is set as 500. First, LSTM [31] and DilatedRNN [32], consisting of cell type as LSTM, set the hidden size to 200 and the context size to 10. Second, NBEATS [33] set the number of harmonic terms for the seasonality stack to 2, the polynomial degree for the trend stack to 2, and the number of hidden layers to 3×(512,512) for each of the three blocks. Third, TCN set kernel size to 2, hidden size to 200, context size to 10, and dilations, that control the time interval between kernel points, to (1, 2, 4, 8, 16). Next, TFT [34] set the hidden size to 64 and the window batch size to 64. Last, PatchTST [35] set the patch length to 4, stride to 4, and window batch size to 512. NeuralProphet [27], STL-based deep learning consisting of additive seasonality, a linear trend, and AR-net was considered. Finally, PaSTANet was separated into AR and STL modules.

For all metrics, PaSTANet exhibited the best performance with 107, 8.63, 96.21, and 81 for MAE, MAPE, SDE, and MedAE, respectively. The Prophet model performed second best with 12.05 and 104.61 for MAPE and SDE, respectively. In MAE, Prophet was the second-best with 152, and in MedAE, PatchTST was the second-best with 112. As a result, the performance evaluation showed that the proposed model has the best performance for all indices (Table 3).

**Table 3:** Comparison of the performances of the proposed and existing models in onion price. The best values are highlighted in bold, and the second-best values are underlined.

Model			MAE (↓)	MAPE (↓)	SDE (↓)	MedAE (↓)
Category	Model Name	Method				
Conventional method	ARIMA [29]	AR	239	18.59	127.74	233
	Prophet [30]	STL	152	12.05	104.61	131
RNN	LSTM [31]	TSF	307	24.71	152.86	338
	DilatedRNN [32]	TSF	268	21.78	148.09	295
FCN	NBEATS [33]	TSF	465	39.02	535.00	264
CNN	TCN [16]	TSF	290	24.34	181.62	330
Transformer	TFT [34]	TSF	392	31.49	220.51	370
	PatchTST [35]	TSF	195	17.48	195.42	112
MLP	NeuralProphet [27]	STL	311	24.26	170.36	359
Proposed	MRCNN	AR	323	26.37	193.71	307
	GaST	STL	278	21.48	150.62	284
	PaSTANet	AR+STL	**107**	**8.53**	**96.21**	**81**

MAE = mean absolute error, MAPE = mean absolute percentage error, SDE = standard deviation error, MedAE = median absolute error, AR = autoregressive, STL = Seasonal and Trend decomposition using Loess, TSF = Time series forecasting, FC = fully connected network, MRCNN = multikernel residual convolution neural network, GaST = Gaussian seasonality trend, PaSTANet = parametric seasonal trend autoregressive neural network.

**Fig 3:**
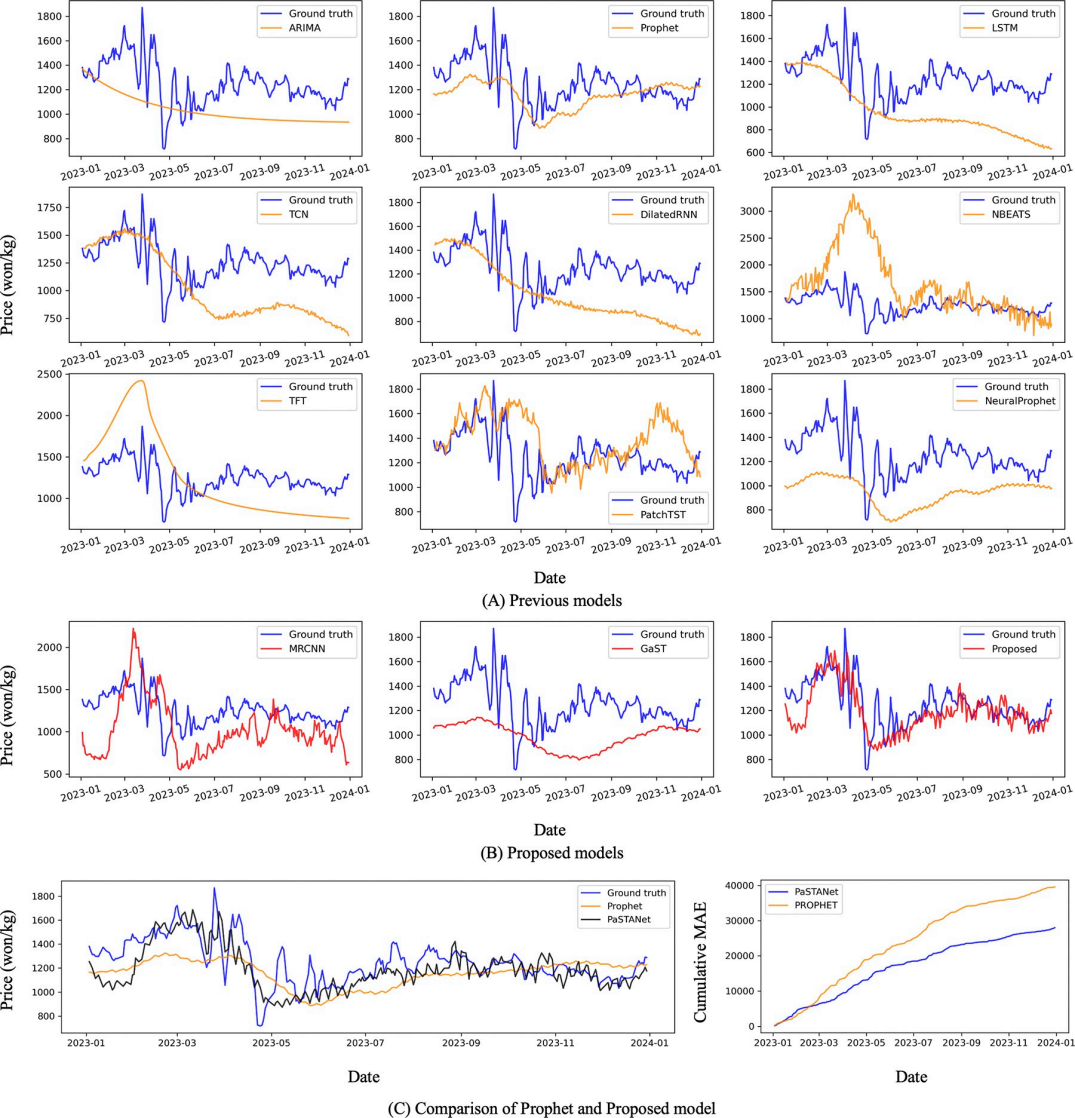
Comparison of model prediction for onion in the test set (2023). (A) The blue line is the actual onion price, and the yellow line is the price predicted by the previous model. (B) The blue line is the actual onion price, and the yellow line is the price predicted by the AR module, STL module, and Parametric Seasonal-Trend Autoregressive Neural network (PaSTANet), respectively. (C) Comparison with actual onion price in the blue line, PaSTANet in the black line and Prophet in the yellow line, and the cumulative mean absolute error (MAE) over time.

The forecasting value and ground truth of the individual models in the test set are displayed in Fig 3. The STL-based models, Prophet and NeuralProphet, provide good fits for the trend and yearly seasonality in forecasting; however, they do not reflect daily volatility. On the other hand, the transformer-based time series forecasting (TSF) models of PatchTST and MRCNN do reflect volatility. Consequently, PaSTANet, which considers both AR and STL, reflects both the trend and volatility in the test set well.

**Fig 4:**
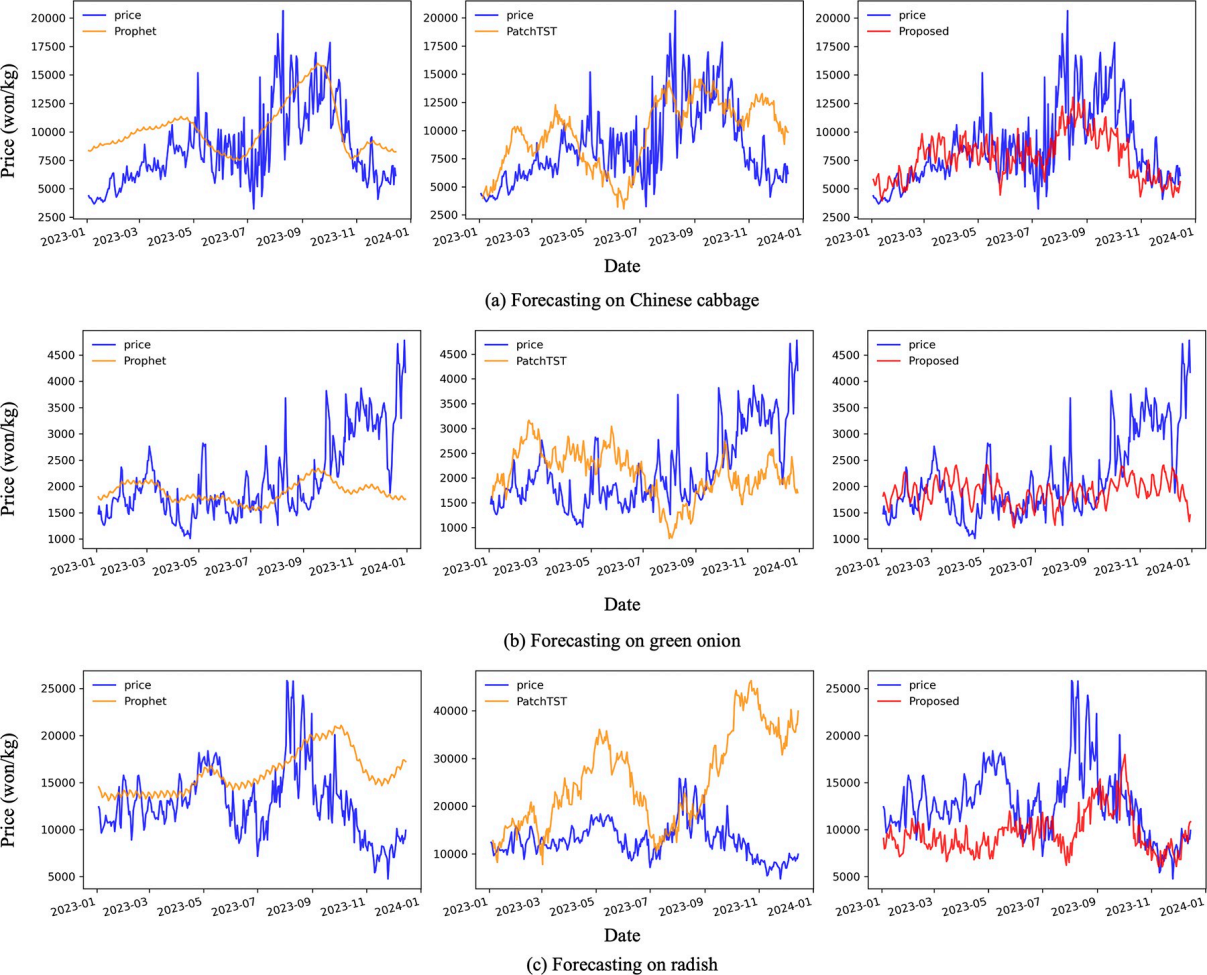
Comparison of model prediction for other crops in the test set. Data from 2023 used for the test set were not used for training and validation. The blue line is the actual price of crops; the orange line is the prediction of previous models; and the red line is the proposed model’s prediction.

We compared the prediction performance of other crop prices with the proposed model using the Prophet model and PatchTST model, which performed well in predicting onion prices (Fig 4). The Prophet model outperformed the SDE for Chinese cabbage and radish and the MedAE for green onion, but the proposed method outperformed all other metrics (Table 4).

**Table 4:** Comparison of performance with existing models and proposed models for cabbage, radish, and green onion. The best values are highlighted in bold.

Crop	Model name	MAE (↓)	MAPE (↓)	SDE (↓)	MedAE (↓)
Chinese cabbage	Prophet	2,677	38.97	**1462.10**	2,767
	PatchTST	2,938	40.97	1983.99	2,771
	PaSTANET	**2,008**	**22.82**	1766.92	**1,505**
Radish	Prophet	3,971	38.40	**2863.55**	3133
	PatchTST	13,055	126.15	10625.59	10538
	PaSTANET	**3,703**	**26.14**	3095.34	**3018**
Green onion	Prophet	570	24.22	558.63	**381**
	PatchTST	783	38.46	551.21	686
	PaSTANet	**557**	**24.19**	**548.56**	382

MAE = mean absolute error, MAPE = mean absolute percentage error, SDE = standard deviation error, MedAE = median absolute error, PaSTANet = Parametric Seasonal-Trend based Autoregressive model.

### 3.2 Abnormal price detection system

**Fig 5:**
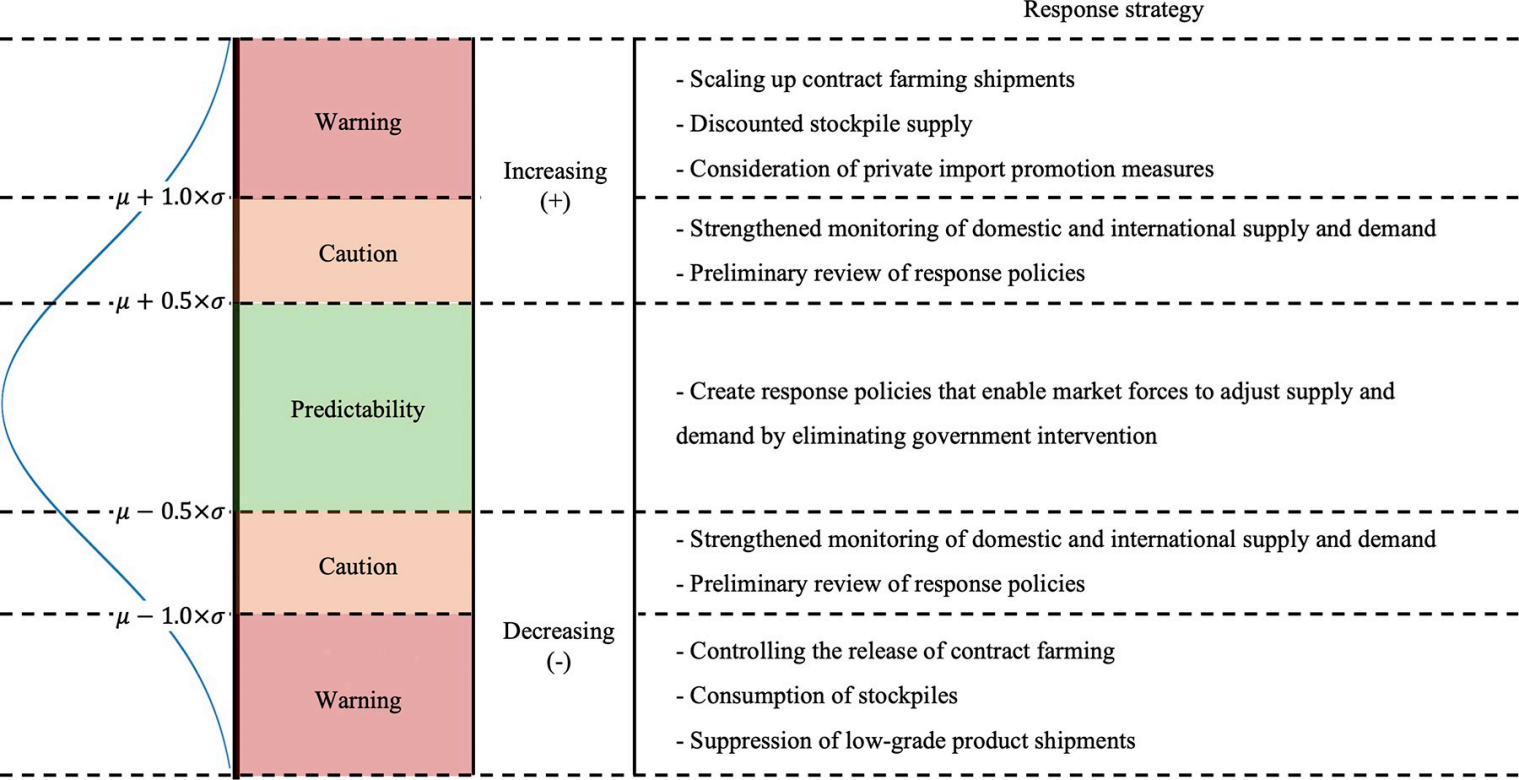
Abnormal price detection system for onions and scenario-based response strategy.

In the long-term forecast of onion prices, recent prices, trends, and seasonality were considered. To propose an appropriate basis for the future pricing of onion, the Gaussian distribution used was divided into three sections according to the confidence interval: predictability, caution, and warning (Fig 5). The criteria that the abnormal price detection system (APDS) uses for each are shown in Fig 5. APDS sets the bands based on the existing agricultural supply and demand control manual, thereby modifying the appropriate bands based on the distribution of forecast values [36]. In the end, we chose to split the bands at 0.5×*σ*^2^ and 1.0×*σ*^2^, as deemed appropriate when comparing producers and consumers given the time of the year that onions are grown.

Table 5 presents the results of applying APDS to the actual prices in the test set of 2023. First, most onion prices are in the predictability zone; however, in January, March, April, and May, the percentage of prices in the caution zone is higher than for other months. In March and May, prices fall into the warning zone. Ultimately, 91.5% of all prices in 2023 are categorized as predictability prices, 8.2% as caution prices, and 0.3% as warning prices.

**Table 5:** The proportion of monthly abnormal price detection for onions in the test set (2023).

Date		PaSTANet			
		Range (min, max)	Predictability (%)	Caution (%)	Warning (%)
Storge of 2022	Jan	(1018, 1254)	69.6	30.4	0.0
	Feb	(1147, 1587)	95.8	4.2	0.0
	Mar	(1316, 1688)	88.9	11.1	0.0
	Apr	(914, 1519)	72.0	28.0	0.0
	May	(853, 1023)	74.1	22.2	3.7
	Jun	(919, 1125)	100.0	0.0	0.0
	Jul	(1054, 1203)	96.2	3.8	0.0
Production of 2023	Aug	(1061, 1422)	100.0	0.0	0.0
	Sep	(1108, 1343)	100.0	0.0	0.0
	Oct	(1077, 1326)	100.0	0.0	0.0
	Nov	(1013, 1253)	100.0	0.0	0.0
	Dec	(1016, 1207)	100.0	0.0	0.0
Total		(853, 1688)	91.5	8.2	0.3

PaSTANet = parametric seasonal-trend autoregressive neural network.

**Fig 6:**
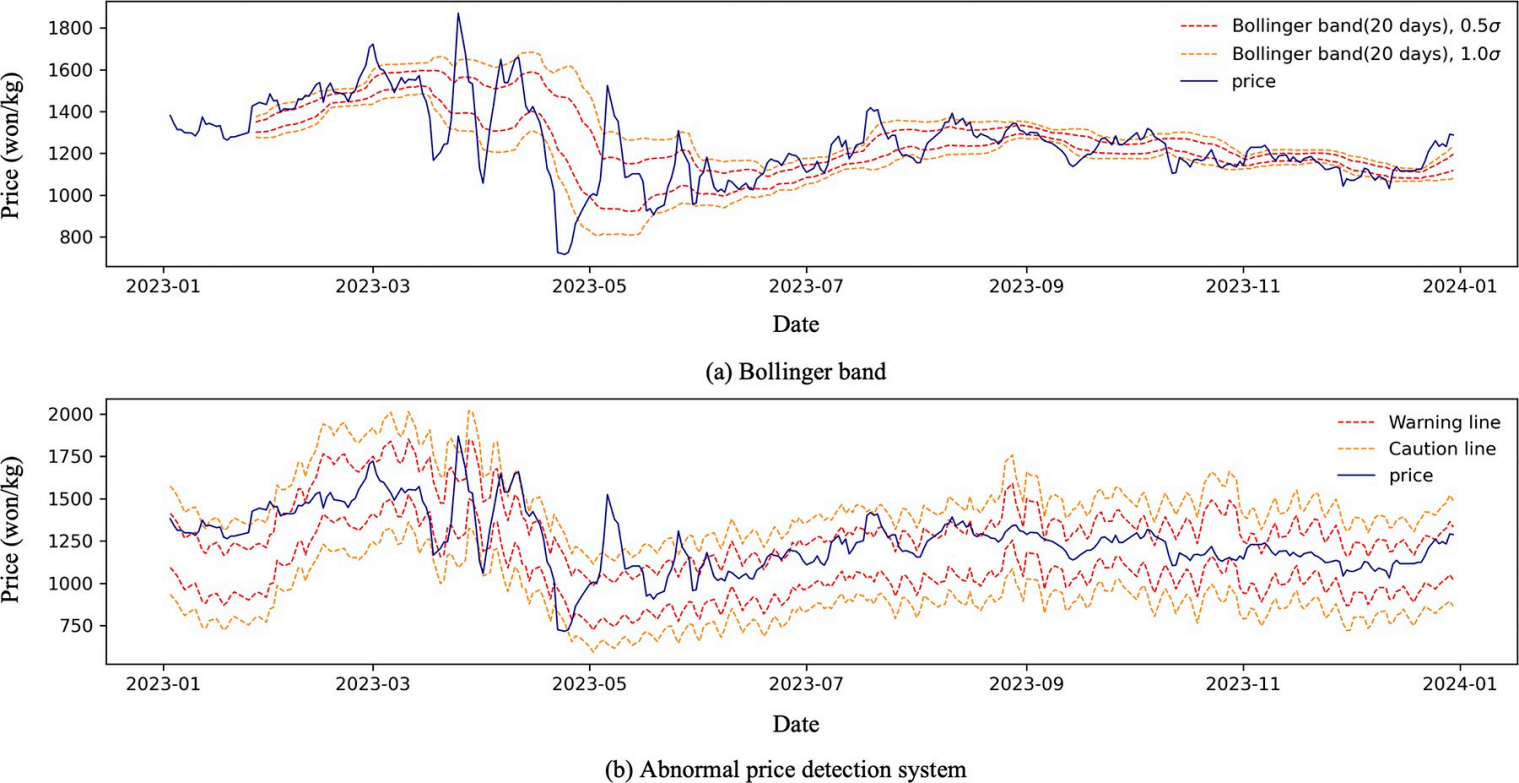
Comparison between Bollinger band and abnormal price detection system.

The APDS was applied to the predicted price, using the estimated variance and mean for binning, which was compared to Bollinger bands (Fig 6). In economy, the Bollinger band is used to separate abnormal prices, using moving average and variance [37–39]. The length of the Bollinger bands’ abnormal price intervals increases during periods of high volatility from March to June and decreases from September onward as fluctuations decrease. APDS considers trends, seasonality as well as recent prices; therefore, the length of the confidence interval is constant.

## 4. Discussion

This study differs from probabilistic sampling models, such as variation autoencoder, in that it directly estimates the mean and variance derived from AR and STL models. This is related to studies that directly estimate the parameters of a distribution, such as regression, which is a conventional model, or mixture density networks [40] using deep learning. While they are not stochastically different from each other in terms of estimating the parameters of the distribution, they can contain uncertainty in that they estimate the variance, which proposed APDS using CI. The study of estimating the parameters of such distributions has been considered in various fields [41–43].

To respond to the impact of onion prices and agricultural crops on the national economy, the price should be forecast daily. Existing studies find it difficult to respond to immediate price volatility because they predict monthly [8,44]. Furthermore, the average price does not fully reflect price volatility. In this study, a forecasting model is proposed using daily onion prices to suggest response strategies.

The AR-based model, commonly used in TSF, has the advantage of being intuitively understandable because it uses recent price information to predict future prices; however, they are sensitive to missing values and outliers, performing poorly in long-term predictions. The crop price data, in particular, has gaps in data during holidays and when no transactions occur, and outliers are inherent for a variety of reasons, including weather. Therefore, the statistical model, the RNN-based and the Transformer-based models, which have shown good performance among TSF models, may not be suitable for this data because the sequence information may be applied irregularly. For example, the *autoregressive integrated moving average* (ARIMA) model has been used for various time-series data; however, it exhibited poor model performance in this study possibly because the lag of AR is difficult to determine because daily data contain missing values due to holidays and absence of transactions, and the rolling forecasting method is used, degrading long-term forecasting performance. The MRCNN module in PaSTANet uses different CNN kernel sizes for the lag to extract weekly and monthly features, subsequently combining them. As a result, the performance was better than those of existing AR models.

The STL models have the advantage of being able to make long-term predictions because they utilize the target feature as a time variable in making predictions; they also have the advantage of being able to bring individual interpretations because they combine trends and seasonality in an additive method. There is a limitation in that long-term predictions are highly affected by trends, making it difficult to reflect recent price information. In addition, the STL model reflects the trend and seasonality of the test set with lower volatility. The GaST module in PaSTANet was converted from a piecewise regression model to a deep-learning-based model. This is an advantage of the deep learning model in that the modules can freely be combined with other models and expanded. Finally, PaSTANet, combines the characteristics of AR and STL, exhibiting superior performance compared to the other models.

The PaSTANet has two main contributions. The first contribution is superior forecasting performance. We compared it to RNN, TCN, and Transformers, which have performed well in various previous time series forecasting studies. However, RNN-based and Transformer models have difficulty determining the baseline of sequence length for crop price data. TCN also suffers from the need to determine the appropriate kernel size for seasonal crop prices. Otherwise, MRCNN can compensate for these problems by enabling parallel sequence processing with multiple kernel sizes that capture patterns at different temporal scales [45]. Furthermore, STL complements this by effectively managing non-stationary data [30]. Therefore, by integrating an MRCNN with STL, we can decompose a time series into its components and leverage STL’s decomposition and CNN’s pattern recognition to efficiently capture complex temporal patterns, model long-term dependencies, and adapt to different temporal scales.

The second contribution of PaSTANet is that it enables the classification of intervals by estimating the Gaussian distribution. The classified predictability, caution, and warning intervals are determined based on the length of the confidence interval, and the risk level is classified based on the interval containing the actual price in the set interval. The price falls into the predictability zone; however, for January, April, March, and May, it falls into the caution zone. The climate of Korea makes January highly volatile because it is the time when onions are produced before they are consumed; March, April, and May are the main production periods for onions. Therefore, the APDS of our model accurately reflects onion production and consumption in Korea.

The PaSTANet output is a Gaussian distribution of prices predicted using recent prices, trends, and seasonality. Therefore, if the actual price deviates from this distribution by a certain amount, it is considered an anomaly. APDS categorizes based on confidence intervals, dividing CI into three stages, and proposing a response strategy for each stage. This means that the existing agricultural supply and demand control manual [36] can overcome the limitation of only reflecting recent prices as the use of daily prices enables detailed weekly, monthly, and quarterly responses.

The ADPS was also compared to Bollinger bands, which have three disadvantages. First, in Bollinger bands, the range of abnormal changes depends on the appropriate window size [46]. Second, the detection of abnormal price performance is degraded in windows that contain high- and low-volatility bins. Finally, Bollinger bands can only be applied to observed values, making it difficult to set long-term bands. In comparison, APDS has the advantage of being able to set bands in advance with a long-term forecast of one year, not requiring a window and considering in addition to recent prices, both trend and seasonality.

The study estimates the distribution of crop prices based on both time and previous prices, to provide a one-year forecast. In future studies, to improve performance, we will consider incorporating information on exogenous variables that can be identified in advance and crops that are substitutes or complements. In addition, the proposed model should be extended to crops.

## 5. Conclusion

Forecasting crop prices are difficult to predict due to the seasonal nature of crops grown in open fields, and fluctuations in prices have a significant impact on the national economy. The PaSTANet proposed in this study estimates the distribution of daily prices using a model that combines MRCNN and GaST. In comparison with various conventional and deep-learning-based models, PaSTANet showed the best performance. In more detail, PaSTANet improved the performance of onion by 29.6%, Chinese cabbage by 25.0%, radishsms by 6.7%, and green onion by 2.2% compared to the Prophet model, which was the second-best performer by MAE. Moreover, we proposed APDS which utilizes a Gaussian distribution.

The proposed model is limited by its inability to incorporate exogenous variables. Because crop prices are also affected by environmental variables and policies, further research on models that include exogenous variables is required. Furthermore, the predicted price is assumed to be normally distributed, but there may be differences in the variance at a particular time, therefore it should be needed to consider a generalized distribution. Finally, information on other crops should be included in the model as there may be complementary or substitutive relationships between crop prices. In future research, a multivariate model will be developed to reflect the complex influences among crops, including also exogenous variables such as weather.
